# Integrating network pharmacology with ex-vivo analysis to assess the effect of IL-2 in halting breast cancer: involvement of Treg/CTLA-4/Blimp-1/caspase-3

**DOI:** 10.1038/s41598-026-52551-2

**Published:** 2026-05-26

**Authors:** Seham Abou Shousha, Sherihan Salaheldin Abdelhamid Ibrahim, Hend Kadry, Eman Ahmed Youssef, Yasmine qabany, Amira A. Darwish, Atef Metwea, Suzan A. Baheeg, Yasmine Shahine

**Affiliations:** 1 Medical Research Institute, Immunology and Allergy Department, Alexandria University, Alexandria, Egypt; 2 Faculty of Pharmacy, Department of Pharmacology & Therapeutics, Pharos University in Alexandria, Canal El- Mahmoudia Street, Smouha, Alexandria, Egypt; 3 Medical Research Institute, Cell Biology Department, Alexandria University, Alexandria, Egypt; 4 Faculty of Allied health sciences technology, Pharos University in Alexandria, Alexandria, Egypt; 5 Medical Research Institute, Department of Surgery, Alexandria University, Alexandria, Egypt; 6 National Institute of Oceanography and Fisheries, Central Laboratories Unit, Alexandria, Egypt; 7 Faculty of Pharmacy, Department of Microbiology & Immunology, Pharos University in Alexandria, Alexandria, Egypt

**Keywords:** Cancer, Immunology, Oncology

## Abstract

The T lymphocytes have a vital role in tumor immunosurveillance within the tumor microenvironment (TME) but on the other hand, the TME adopts several mechanisms that cause inhibition and apoptosis to effector T cells leading to tumor immune evasion. The controversial role of interleukin-2 (IL-2) was demonstrated by promoting the activation and proliferation of different immune cells such as NK cells, effector and regulatory T cells (T regs). Thus, ex-vivo approach was used to investigate IL-2 anti-tumor effect on breast cancer cells isolated from Egyptian patients after mastectomy via modulating Treg/CTLA-4/Blimp-1/caspase-3 trajectory. Previous to the ex-vivo approach we aimed to find common targets between IL-2 and breast cancer disease using network pharmacology. Results of network pharmacology illustrated that there were 35 common targets including CD4, CTLA-4 and caspase 3. Breast cancers cells obtained were then cultured in the presence of 10 µl of (50 ng/ml) recombinant IL-2 for 24 h. Results revealed that tissue culture supplementation with IL-2 significantly reduced T regs within the TME via inhibiting the tumor expression of CD25 and Forkhead box P3 (FOXP3). In addition, it was found that IL-2 significantly decreased the expression of the inhibitory receptor CTLA-4 and increased the expression of B lymphocyte–induced maturation protein-1(Blimp-1), leading to the activation of effector T cells and the induction apoptosis of the breast tumor cells. These findings suggest that IL-2 could serve as a new treatment strategy for breast cancer via modulating immune responses within the TME.

**Supplementary Information:**

The online version contains supplementary material available at 10.1038/s41598-026-52551-2.

## Introduction

In cancers, a variety of tumor cells and non-cancerous cells are lodged in a modified extracellular matrix to form complex ecosystems. The effectiveness of immunotherapeutic approaches in cancer treatment is significantly influenced by the tumor microenvironment (TME). The TME is made up of tumor cells, immune cells that infiltrate tumors, stromal cells, and other cellular and molecular components. Also, it is hypoxic and rich in growth factors that support angiogenesis and immune evasion^1^.

Immune-suppressive cells, particularly regulatory T cells (Tregs), play a crucial role in maintaining immune homeostasis and preventing autoimmunity. The development and function of CD4 Tregs are dependent on the transcription factors Forkhead box P3 (FOXP3) and the IL-2 receptor α chain (CD25)^2^. Approximately, 5% of circulating CD4 T lymphocytes in the peripheral blood are (Tregs), which consist of different cell subsets^3^. Natural Tregs (nTregs), generated from the thymus, maintain immunological homeostasis, while inducible Tregs (iTregs) respond to external stimuli and help regulate T helper responses during inflammation. Furthermore, the iTregs are also significant in pathological conditions, including cancer immune evasion. Whiteside^4^,. The Tregs impair anti-tumor immunity and facilitate cancer progression via weakening functions of effector T cells (Teffs) via utilizing immune checkpoint receptors like cytotoxic T-lymphocyte-associated antigen 4 (CTLA-4) (Zhang, A et al.^5^, and^6^and Westendorf AM et al.^7^,). Moreover, The B lymphocyte-induced maturation protein-1 (Blimp-1) is a critical regulator that helps maintain the suppressive activity of Tregs within the TME thus could inhibit the invasion of breast cancer cells. (Chen YF et al.^8^, and^9^.

The main source of the pleiotropic interleukin 2 cytokine (IL-2) is antigen-stimulated CD4 T cells. Other sources include activated dendritic cells, NK cells, and CD8 T cells. It was shown that IL-2 primarily promotes NK cytolytic activity, and T-cell proliferation thus inducing programmed cell death, and on the other hand, it has a vital role in regulatory T-cell differentiation^10^. Low, intermediate, and high-affinity IL-2 receptors are formed via three distinct IL-2 receptor chains. Activated T cells and NK cells express the ligand-specific IL-2 receptor α chain (CD25), which binds IL-2 with low affinity; IL-2Rβ (CD122) and IL-2Rγc (CD132) together form an IL-2Rβ/γc complex, which binds IL-2 with intermediate affinity primarily on memory T cells and NK cells; and IL-2 is bound with high affinity when all three receptor chains are co-expressed on Treg cells and activated T cells (Kahan SM et al.^11^).

Multiple studies reported that IL-2 can have both anti-tumor and tumor-supporting action mainly based on its concentration. Thus, we aimed to investigate its anti-tumor effect in breast cancer ex-vivo via highlighting its effect on CD4 T cells, Tregs, CTLA-4 and Blimp-1 expression in Egyptian breast cancer patients^12^. Previous to ex-vivo study, network pharmacology was used. Network pharmacology offers a smart, systems-based strategy to illustrate “common targets,” between the IL-2 and breast cancer disease. These prioritized targets then undergo experimental validation (ex-vivo) to confirm how the IL-2 actually interferes with disease processes. This blend of computational prediction and ex-vivo results accelerates the discovery of mechanisms and increases the chances of finding effective, multi-target therapies **(**Alotaibi, N. M et al.^13^).

## Subjects & methods

### Network pharmacology approach for screening common targets for both IL-2 and breast cancer disease

#### Genetic mapping of breast cancer

Gene cards database was used to identify the target genes of breast cancer (https://www.genecards.org/**).**

#### Targets prediction of IL-2

The IL-2 chemical structure was identified by **Pubchem**. Furthermore, IL-2 protein targets were mapped from numerous databases to ensure complete coverage of all relevant genes. These databases include, Swiss Target Prediction **(**http://www.swisstargetprediction.ch/**)**, TargetNet **(**http://targetnet.scbdd.com/calcnet/index/**).**

#### Networks construction and analysis

Common genes to breast cancer and IL-2 were identified by Venny 2.1 **(**https://bioinfogp.cnb.csic.es/tools/venny/**).** Then, Cytoscape 3.10 software and STRING version 12 database **(**https://string-db.org/**)** were used to construct breast cancer-gene network, IL-2 -target and Merged network for both disease and IL-2. Moreover, analysis of merged network was done.

### Subjects

The ethical committee at Alexandria University’s medical research institute in Egypt gave its approval to the current study (**E/C. S/N. T81/2017**), which complies with the Declaration of Helsinki’s guidelines. Informed consent was obtained from all individual participants included in the study.

Twenty Egyptian patients with histologically confirmed breast cancer who were scheduled for a modified radical mastectomy were recruited from the Medical Research Institute’s Experimental and Clinical Surgery department at Alexandria University. Individuals with immunologic-mediated diseases, immunocompromised patients, and those who had already received chemotherapy were not included. Clinicopathological data of the studied patients included as (supplementary material 1).

### Methods

#### Tissue cultures of breast tumor and normal tissues

Following surgical dissection, tissue samples of the main breast tumor were taken from each patient, according to the approved medical ethics of Alexandria University. Each tumor sample was separated into two sections: one section was kept on ice in an organ transit medium until it was used for tissue culture, and the other section was used for standard histopathological, immunohistochemical and western blot investigations. After that, the tissues from the breast tumor were imbedded in full RPMI medium (Lonza, Belgium). Equal amounts of each tumor tissue sample were cultured in a 96-well tissue culture plate with and without 10 µl of recombinant IL-2 (**Code: 11011456001**,** ROCHE Interleukin-2 human (hIL-2) recombinant (E. coli)**) (**TT & T**,** respectively**) at a concentration of 50 ng/ml. Liu^14^.

Same procedure was done using peritumoral normal tissue from the same excised breast (**NT & N**). Following that, tissue-cultured samples were incubated for 24 h at 37 °C in a constant atmosphere with 5% CO2. (15) Following the incubation time, the tumor and healthy cultured tissues were preserved for 24 h in 10% phosphate-buffered formalin (PH 7.4) before being processed to create microscope slides for immunological analysis^15^.

#### Immunofluorescence detection of CD4 and CD25 expression

The slides were placed in retrieval sodium citrate buffer 0.01 M, pH: 6.0, heated to 100 °C, and then rinsed with PBS after tissue slices had been deparaffinized, rehydrated, and treated in 3% hydrogen peroxide block H (**Cat#426000250**,** 3 wt% solution in water**,** stabilized**,** Thermo-Scientific Chemicals**,** USA**). To prevent the antibodies from binding unspecifically, the slides were then treated for 30 min with 1% bovine serum albumin (Euroclone, Italy) in phosphate buffer saline. In the same tube, the two primary antibodies were combined at the proper dilutions in PBS (CD4 at 1:200 (**Cat #14–0049−82**,** eBioscience**,** UK**), CD25 at 1:50 (**Cat #14–0256−82**,** eBioscience**,** UK**), applied to the tissues, and then incubated at 4° C for the entire night.

The secondary antibodies (**Cat#405418**,** Alexaflour 488- conjugated anti-rat IgG**,** Jackson Laboratories**,** USA**) and (**Cat#405326**,** Alexaflour 594- conjugated anti mouse IgG**,** Jackson Laboratories**,** USA**) were mixed and diluted in PBS (1:300). The mixture was then added to the sections and the slides were incubated in the dark for 60 min and then washed twice in PBS for 5 min. Finally, the slides were mounted by fluorescent mounting medium containing DAPI (**eBioscience**,** UK**) and examined by fluorescent microscope. Visual assessment of the immunofluorescence stain was done by examining the slides using correct excitation and filters on a fluorescence microscope. The slides were then photographed and the fluorescence intensity of the stain was calculated using ImageJ photos analyzing software^15^.

Photomicrographs of randomly selected non overlapping high powered fields were captured for each tissue section, via using a LSM 510 confocal microscope (**Zeiss**,** Jena**,** Germany**) with a 400 objective. Images were viewed using LSM Image Browser software at a screen resolution of 1,280 by 1,024 pixels. T cells were analyzed for the presence or absence of CD4^+^ and CD25^+^ stained cells within identically photographed fields. Cells were considered positive for CD4^+^ and CD25^+^ based on the presence of intense circular membranous staining. The merged fluorescence intensity of these cells in breast tumor specimens was used to calculate the amount of CD4^+^ and CD25^+^T cells (in merged photomicrographs) (Siddiqui SA et al.^16^).

#### Western blot analysis for determination of FOXP3, CTLA-4 and Blimp-1 expression in normal and tumor breast TME

Fixed paraffin-embedded tissue samples were first deparaffinized and rehydrated using xylene and a series of descending alcohol concentrations, followed by rinsing with PBS. Protein extraction was performed using the Qproteome FFPE Tissue Kit (**Cat. No. 37623**,** Qiagen**,** Italy**). The samples were incubated in an extraction buffer at 100 °C for 20 min and then at 80 °C for 2 h, with final cooling to 4 °C. The supernatant was collected in a new tube.

The extracted proteins were separated using a 10% polyacrylamide SDS-PAGE gel in a Bio-Rad Mini Protean Tetra Cell System at 150 V for 1 h. Protein quantification was carried out using the Bio-Rad Protein Assay Kit, following the manufacturer’s instructions. The separated proteins were transferred onto a nitrocellulose membrane, which was then blocked at room temperature for 1 h in TBST buffer (100 mM Tris, pH 7.5, 0.9% NaCl, 0.1% Tween 20) containing 5% non-fat dry milk. The membranes were incubated overnight at 4 °C with primary antibodies for FOXP3(**Cat# PA5-12396**,** Thermo-Fisher Scientific**,** USA**), CTLA-4 (**Cat# H00001493-M33**,** Thermo-Fisher Scientific**,** USA**), and Blimp-1 (**Cat# 14–5963−82**,** Thermo-Fisher Scientific**,** USA**).

After incubation, the membranes were washed three times with TBST and treated with a goat anti-rabbit HRP-conjugated secondary antibody (**Cat#31460**,** Thermo-Fisher Scientific**,** USA**) at a dilution of 1:20,000. Immunolabeled proteins were detected using ECL substrate, and the chemiluminescent signals were captured using a CCD camera-based imaging system. Protein bands were analyzed with ImageJ software, where the intensity of target protein bands was normalized to beta-actin and compared to the test sample (Lepore MT et al.^17^).

#### Immunohistochemical detection of Caspase-3

Tissue sections were deparaffinized, and rehydrated. Then, slides were incubated in 3% Hydrogen Peroxide block H (**Cat#426000250**,** 3 wt% solution in water**,** stabilized**,** Thermo-Scientific Chemicals**,** USA**). Subsequently, placed in retrieval sodium citrate buffer 0.01 M, pH: 6.0 and heated at 100 °C then washed with PBS. They were incubated with ultra-V block (**Cat#TA-060-UB**,** Thermo-Scientific Chemicals**,** USA**) diluted 1:5 in PBS for 30 min to reduce the nonspecific binding of the conjugated secondary antibody.

The sections were then incubated with “Rabbit polyclonal antibody for caspase-3 (**Cat# ab90437**,** Abcam**,** USA**) (5µg/ml) at 4 °C in a humid chamber. Washed and incubated with Biotinylated goat anti-polyvalent secondary antibody (**Thermo-Biochemicals**,** USA**) for 10 minutes at room temperature. Washed again and Streptavidin peroxidase (**Thermo-Biochemicals**,** USA**) was applied for 10 minutes at room temperature.

One drop (40 µl) of DAB plus chromogen (**Skytek Laboratories**,** USA**) was added to 2 ml of DAB Plus substrate (**Skytek Laboratories**,** USA**), mixed by swirling, and applied to the tissue. The slides were then incubated for 5–15 min, depending on the desired stain intensity. The slides were finally counterstained in Mayer’s hematoxylin and covers lipped using a permanent mounting media.

The DAB chromogen yielded a brown color reaction end product at the site of the target antigen, the immunostaining was expressed according to a semi-quantitative scale. The tumor samples were graded as negative when there was a complete absence of nuclear staining. The positive tumor samples were graded as negative (0) weak (1), moderate (2), and strong (3) according to the degree of nuclear and cytoplasmic staining (Gaber DM et al.^18^).

#### Histopathological assessments

Paraffin-embedded fixed sections from cultured breast tissue of different groups were stained with H&E, and examined by light microscope for assessment of histopathological changes.

#### Statistical analysis

Data were fed to the computer and analyzed using IBM SPSS software package version 20.0 **(**Armonk, NY: IBM Corp**)**. Qualitative data were described using number and percent. The Kolmogorov-Smirnov test was used to verify the normality of distribution.

Quantitative data were described using mean ± standard deviation (SD). Significance of the obtained results was judged at the 5% level. Paired t-test was used for normally distributed quantitative variables, to compare between two studied groups. Correlations between variables were assessed.

## Results

### Network pharmacology approach results

**Fig. 1:**
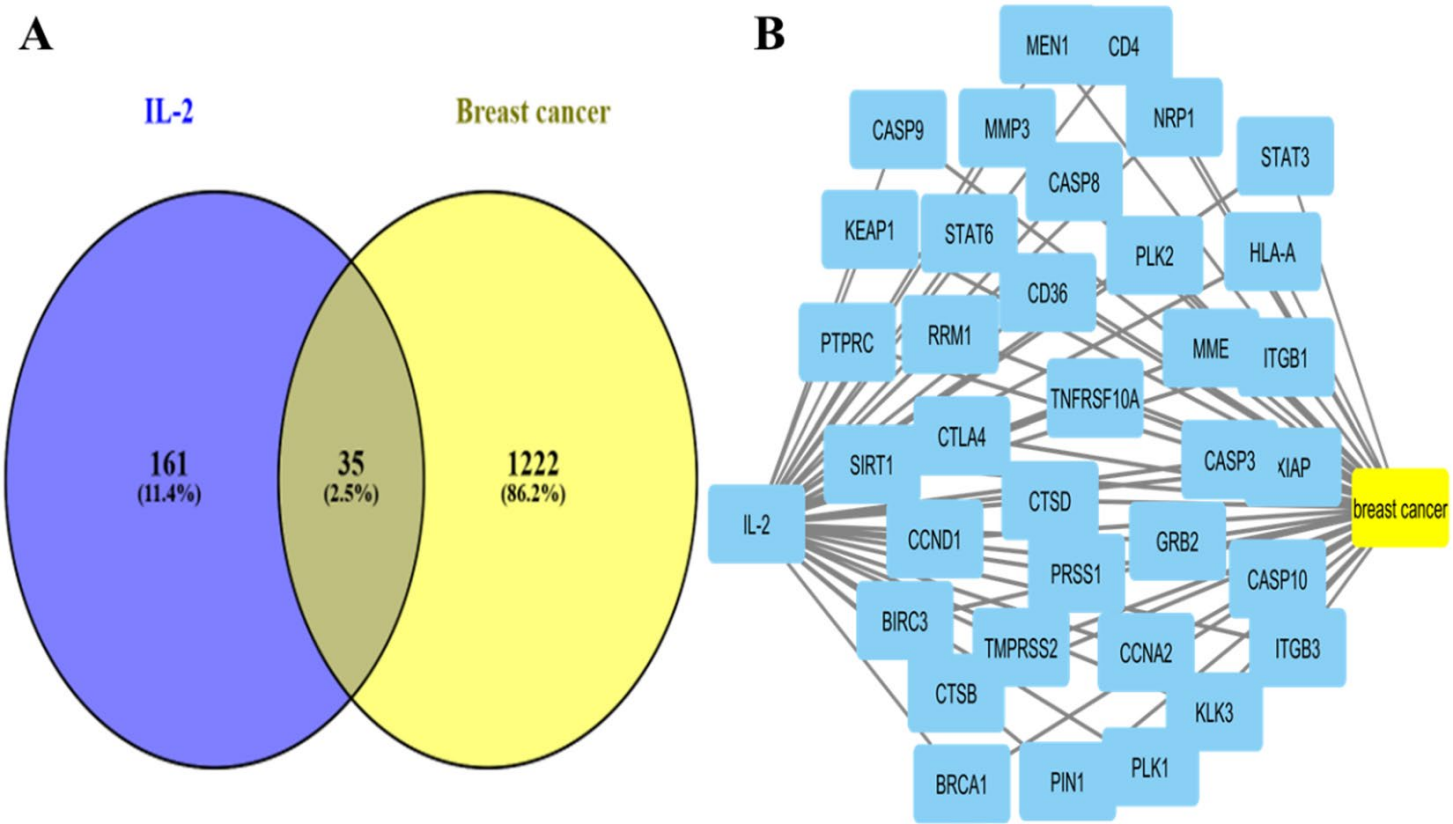
Venn Diagram showing common genes between breast cancer (n = 1222) and IL-2 (n = 161). A total of 35 common targets were identified. (B) Protein-protein interaction (PPI) of 35 intersection targets constructed using STRING database and visualized using Cytoscape.

The target genes of breast cancer were identified by gene cards database; they were 19,018 genes. then 1257 genes were selected with relevance score ≥ 24. Additionally, IL-2 associated genes were predicted using different databases yielding (196 genes). Venny 2.1 revealed that breast cancer and IL-2 possessed 35 common genes (Fig. 1 A). These identified common genes were then utilized to build the Breast cancer-target-IL-2 network (Fig. 1B; Table 1).

**Table 1:** Common targets between IL-2 and breast cancer:.

IL-2/Breast cancer	Common targets (symbol)	Common targets (Full name)
	BIRC3	baculoviral IAP repeat containing 3
	BRCA1	BRCA1 DNA repair associated
	CASP10	caspase 10
	CASP3	Caspase 3
	CASP8	caspase 8
	CASP9	caspase 9
	CCNA2	cyclin A2
	CCND1	cyclin D1
	CD36	CD36 molecule
	CD4	CD4 molecule
	CTSB	cathepsin B
	CTSD	cathepsin D
	GRB2	growth factor receptor bound protein 2
	HLA-A	major histocompatibility complex, class I, A
	ITGB1	integrin subunit beta 1
	ITGB3	integrin subunit beta 3
	KEAP1	kelch like ECH associated protein 1
	KLK3	kallikrein related peptidase 3
	MEN1	menin 1
	MME	membrane metalloendopeptidase
	MMP3	matrix metallopeptidase 3
	NRP1	neuropilin 1
	PIN1	peptidylprolyl cis/trans isomerase, NIMA-interacting 1
	PLK1	polo like kinase 1
	PLK2	polo like kinase 2
	PRSS1	serine protease 1
	PTPRC	protein tyrosine phosphatase receptor type C
	RRM1	ribonucleotide reductase catalytic subunit M1
	SIRT1	sirtuin 1
	STAT3	signal transducer and activator of transcription 3
	STAT6	signal transducer and activator of transcription 6
	TMPRSS2	transmembrane serine protease 2
	TNFRSF10A	TNF receptor superfamily member 10a
	XIAP	X-linked inhibitor of apoptosis
	CTLA4	cytotoxic T-lymphocyte associated protein 4

### Ex-vivo approach analysis

#### Immunofluorescence detection of CD4 and CD25 expression

No significant difference was noticed between the CD4 expression within the control tumor and normal tissues (T & N). In contrast, a significant difference was detected in the co-expression of CD4 & CD25 in those groups (T & N).

Moreover, CD4 expression and co-expression of CD4 & CD25 were significantly decreased in the tumor tissue culture system supplemented with recombinant IL-2 (T + IL-2) as compared to the tumor tissue without supplementation (T).

**Fig. 2:**
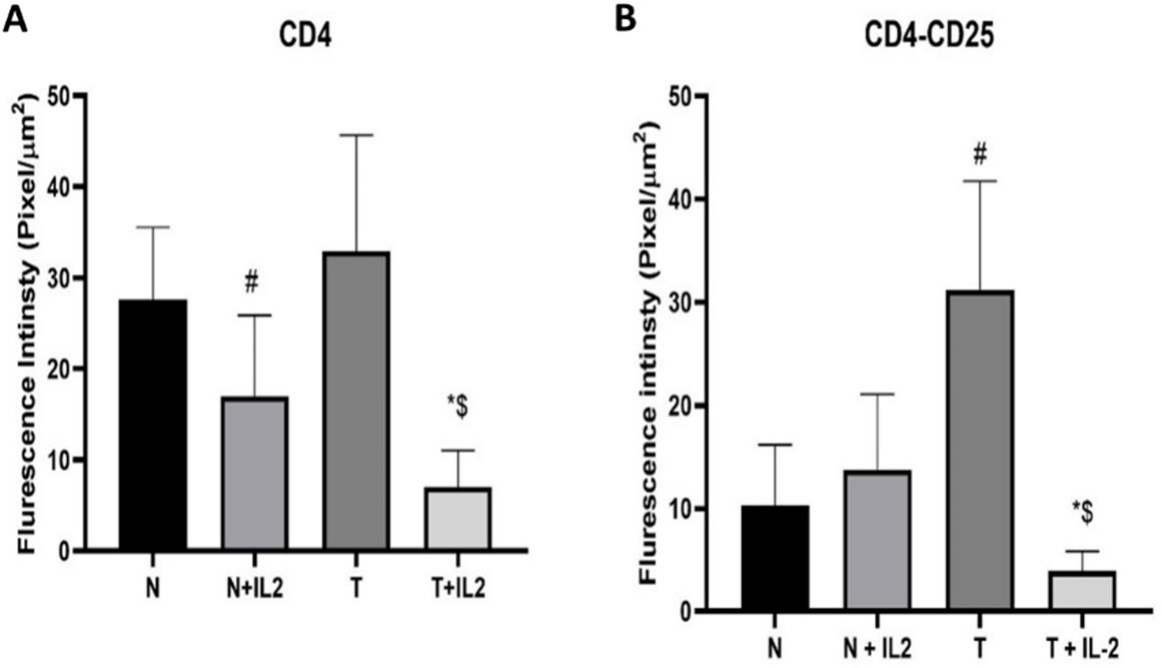
Fluorescence intensity of both CD4 Expression and CD4/CD25 co-expression in the different tissue culture systems. (**A)**: CD4 expression intensity (**B)**: CD4/CD25 co-expression intensity. Data presented as mean ± SD. # significant from N, * Significant from T, $ significant from N+IL2.

**Fig. 3:**
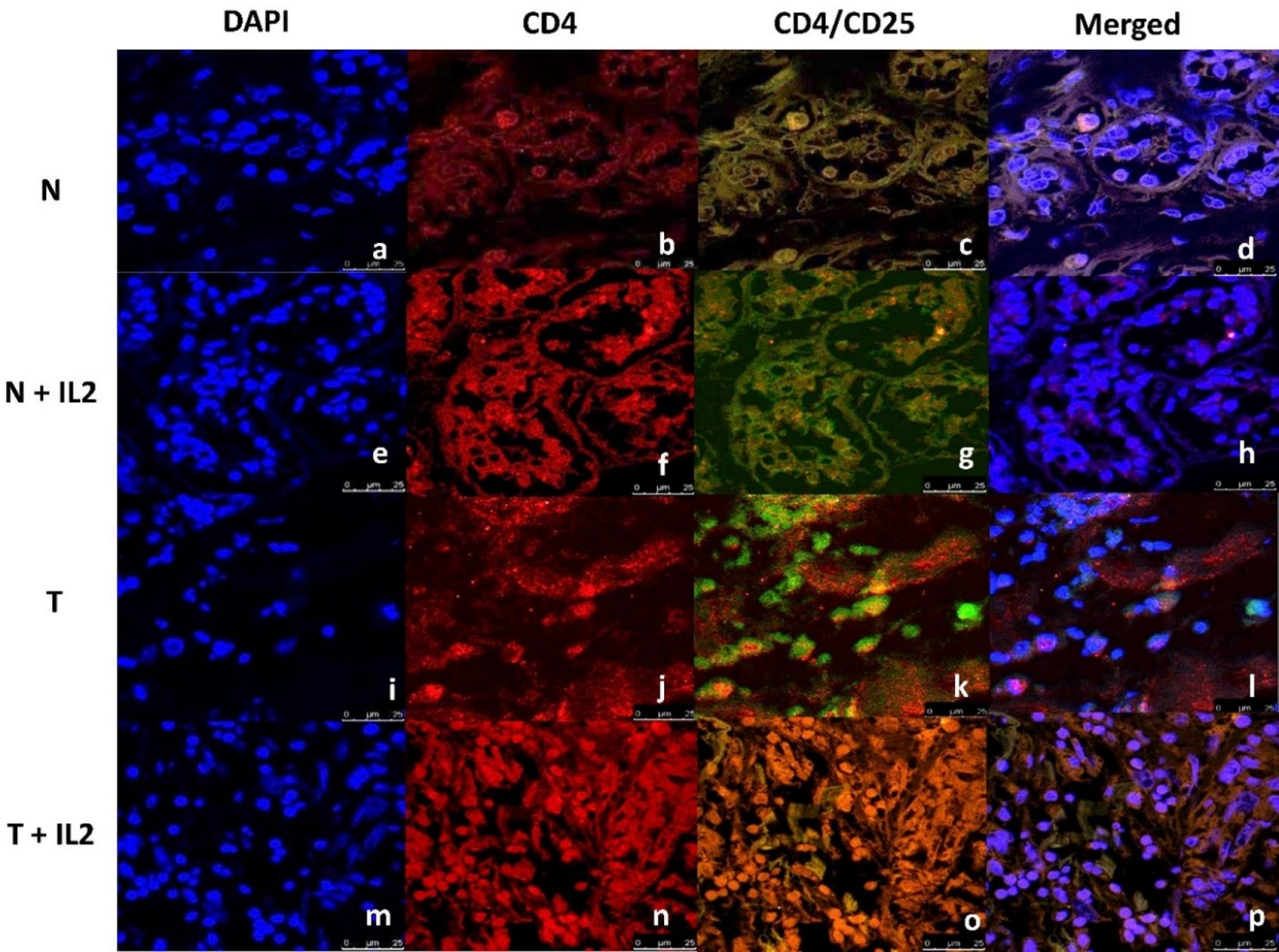
Immunofluorescence staining of normal and tumor breast tissues treated or untreated with recombinant IL-2. (**a**,** e**,** i**,** m**): DAPI nuclear staining (Blue) in the different tissue cultures (N, N+IL2, T, T+IL2), respectively (Confocal microscopy, magnification ×600). (**b**,** f**,** j**,** n**): CD4 expression in the different cultures (N, N+IL2, T, T+IL2), respectively, showing fluorescence intensity for Alexafluor 594 staining (red) (Confocal microscopy ×600). (**c**,** g**,** k**,** o**): CD4/CD25 expression in the different tissue cultures (N, N+IL2, T, T+IL2), respectively, showing merged fluorescence intensity (Confocal microscopy, magnification ×600). (**d**,** h**,** l**,** p**): CD4/CD25 expression merged images in the different tissue cultures (N, N+IL2, T, T+IL2), respectively (Confocal microscopy, ×600).

In contrast, both CD4 expression and CD4/CD25 co-expression were significantly less in the tumor tissue culture supplemented with recombinant IL-2 (T + IL2) compared to its normal counterpart (N + IL2), (Table 2, and Figs. 2 and 3).

**Table 2:** CD4 & CD4/CD25 co-expression in the different designed tissue culture systems.

CD4 Expression					
	*N* (n = 20)		*N* + IL-2 (n = 20)	**T** (n = 20)	**T + IL-2** (n = 20)
Mean ± SD	27.60 ± 7.94		17.03 ± 8.85	32.93 ± 12.76	7.0 ± 4.03

Co-expression of CD4 & CD25					
	**N** (*n* = 20)	**N + IL-2** (*n* = 20)		**T** (*n* = 20)	**T + IL-2** (*n* = 20)
Mean ± SD.	10.32 ± 5.89	13.75 ± 7.33		12.55 ± 5.77	3.94 ± 1.90

#### Correlation between the level of CD4 + cells and CD4+/CD25 + in breast tumor tissue cultures with the clinicopathological parameters among breast cancer patients

There was a significant correlation between the level of CD4 expression in the IL-2-treated breast tumor tissue and the tumor size. Tumor sizes with less than 2 cm (T1) have significantly higher CD4 T cells (mean 11.16 ± 5.18) (*p* = 0.019).

In addition, there was a significant correlation between the level of CD4 expression in the IL-2-treated breast tumor tissue and the stage of the breast tumor of the patients. Tumors with stage I have a significantly higher mean of CD4^+^ cells (14.30 ± 3.10) (*p* < 0.001). No significant correlations have been found with the rest of the parameters (Table 3).

Moreover, there was a significant correlation between the level of CD4/CD25 expression in the IL-2-treated breast tumor tissue cultures and the tumor stage and the tumor size of the studied patients. The mean number of Tregs were significantly higher in tumor sizes more than 2 cm (*p* = 0.039) and in late stages (Stage II and III) (*p* = 0.026). However, there was an insignificant correlation between the level of CD4/CD25 expression in the IL-2 treated breast tumor tissue cultures with age, lymphovascular involvement, vascular invasion, and the hormonal status of the studied patients. (Table 4)

**Table 3:** Correlation between the CD4 expression levels in IL-2 treated breast tumor tissue cultures and different clinicopathological parameters.

	CD4 expression levels in IL-2 treated breast tumor tissue cultures			Test of Sig.	*P*
	Min. – Max.	Mean ± SD.	Median		
Age (years)					
< 50	3.78–12.29	6.46 ± 3.26	5.52	t = 0.383	0.706
≥ 50	2.19–17.87	7.23 ± 4.41	7.28		
**V inv.**					
Negative	2.47–12.74	6.72 ± 4.54	5.84	t = 0.152	0.881
Positive	2.19–17.87	7.07 ± 4.05	6.99		
**LN inv.**					
Negative	2.47–17.87	9.48 ± 5.86	9.83	t = 1.420	0.207
Positive	2.19–10.79	5.94 ± 2.55	5.93		
**ER**					
Negative	2.85–6.79	4.82 ± 2.78	4.82	F = 0.969	0.431
+	3.78–4.10	3.94 ± 0.23	3.94		
++	2.19–17.87	8.96 ± 6.20	7.37		
+++	2.47–12.74	7.07 ± 3.15	7.58		
**PR**					
Negative	2.85–6.79	4.82 ± 2.78	4.82	F = 0.557	0.651
+	2.19–12.29	5.95 ± 4.02	4.10		
++	3.87–17.87	8.23 ± 4.48	7.58		
+++	2.47–10.79	6.65 ± 3.94	6.67		
**T**					
1	3.78–17.87	11.16 ± 5.18	12.29	F = 5.065^*^	0.019^*^
2	2.19–10.79	5.71 ± 3.12	5.08		
3	2.85–7.77	5.54 ± 1.98	5.48		
**N**					
0	2.47–17.87	9.48 ± 5.86 6.05 ± 3.09 6.83 ± 1.78 4.82 ± 2.78	9.83	F = 1.058	0.396
1	2.19–10.79		5.08		
2	4.16–7.82		7.67		
3	2.85–6.79		4.82		
**Stage**					
I	12.29–17.87	14.30 ± 3.10	12.74	F = 14.040^*^	< 0.001^*^
II	2.19–10.79	5.30 ± 2.86	4.17		
III	2.85–7.82	6.31 ± 1.98	7.19		

t: Student t-test, F: F for ANOVA test, p: p value for association between different categories *: Statistically significant at p ≤ 0.05.

**Table 4:** Correlation between the CD4/CD25 expression levels in IL-2 treated breast tumor tissue cultures and different clinicopathological parameters of the studied patients.

	CD4/CD25 expression levels in IL-2 treated breast tumor tissue cultures			Test of Sig.	*P*
	Min. – Max.	Mean ± SD.	Median		
Age (years)					
< 50	1.0–7.0	3.96 ± 2.06	3.96	t = 0.024	0.981
≥ 50	1.67–7.76	3.93 ± 1.92	3.57		
**V inv.**					
Negative	1.93–7.0	3.76 ± 2.38	3.06	t = 0.205	0.840
Positive	1.0–7.76	3.98 ± 1.86	3.91		
**LN inv.**					
Negative	1.93–5.16	3.16 ± 1.26	2.88	t = 1.211	0.241
Positive	1.0–7.76	4.27 ± 2.07	4.33		
**ER**					
Negative	2.50–5.16	3.83 ± 1.88	3.83	F = 0.681	0.576
+	1.0–4.10	2.55 ± 2.19	2.55		
++	1.67–5.16	3.45 ± 1.44	3.10		
+++	1.93–7.76	4.43 ± 2.11	4.01		
**PR**					
Negative	2.50–5.16	3.83 ± 1.88	3.83	F = 1.169	0.353
+	1.0–5.16	2.92 ± 1.71	2.66		
++	1.93–7.76	4.76 ± 1.97	4.64		
+++	2.0–5.86	3.42 ± 1.84	2.91		
**T**					
1	1.0–3.10	2.14 ± 0.80	2.0	F = 3.943^*^	0.039^*^
2	1.67–7.76	4.42 ± 2.14	4.64		
3	2.50–7.0	4.65 ± 1.55	4.44		
**N**					
0	1.93–5.16	3.16 ± 1.26	2.88	F = 1.995	0.158
1	1.0–6.50	3.64 ± 2.15	3.81		
2	4.01–7.76	5.89 ± 1.78	5.89		
3	2.50–5.16	3.83 ± 1.88	3.83		
**Stage**					
I	1.93–3.10	2.56 ± 0.59	2.66	F = 4.544^*^	0.026^*^
II	1.0–5.86	3.34 ± 1.63	3.47		
III	2.50–7.76	5.39 ± 1.83	5.16		

t: Student t-test, F: F for ANOVA test, p: p value for association between different categories, *: Statistically significant at p ≤ 0.05.

#### Western blot analysis

**Fig. 4:**
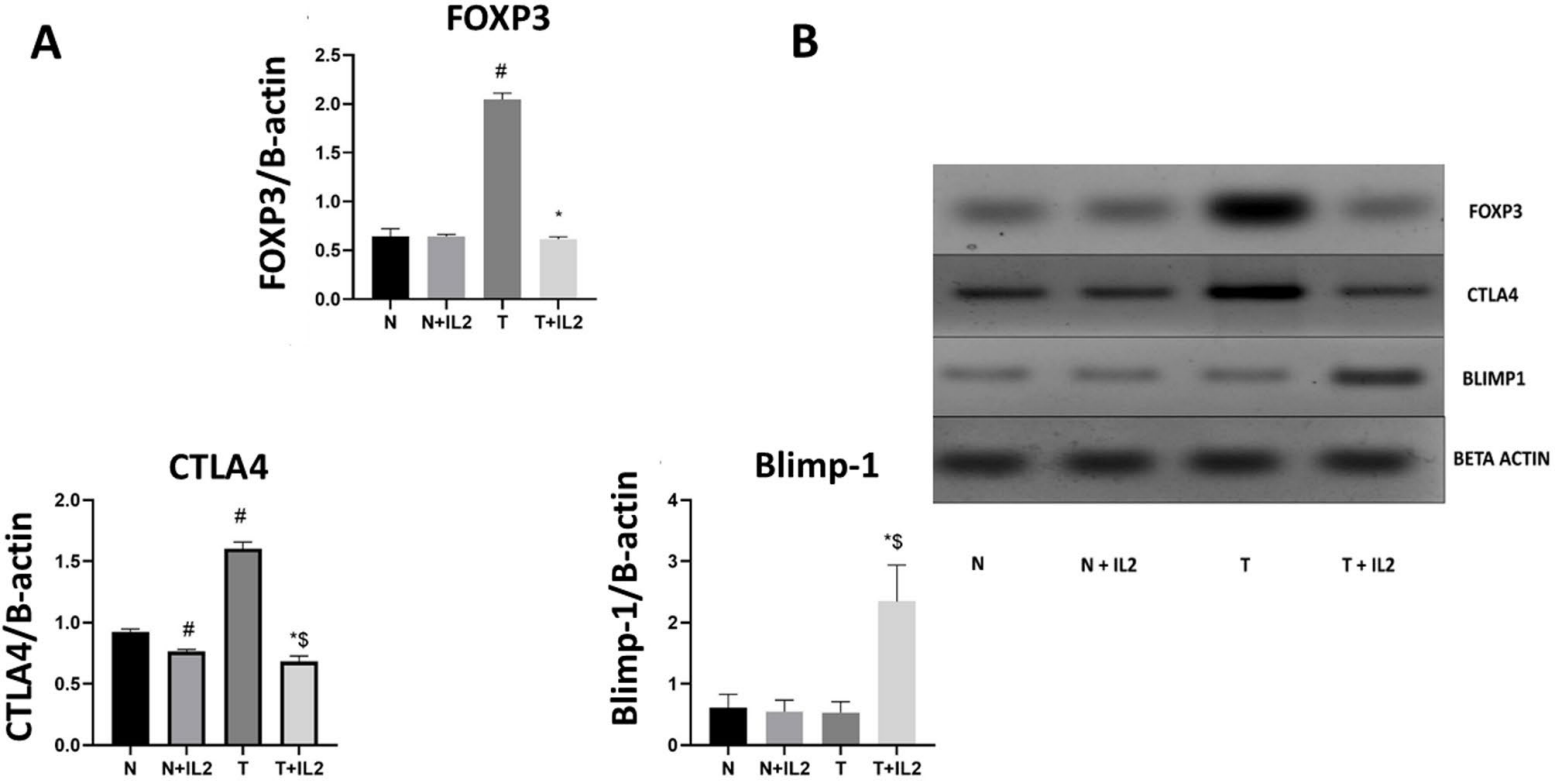
Western blot analysis for determination of FOXP3, CTLA-4 and Blimp-1 expression in normal and tumor breast tissues. (**A**): Bar chart for protein relative expression, (**B**): Western blot expression bands of assessed proteins of different groups. # significant from N, * Significant from T, $ significant from N+IL2.

Western blot analysis was done to detect the expression of different immune parameters to reveal the effect of IL2 on the link between the regulatory T lymphocytes and the effector T cells, (Fig. 4). Results revealed that FOXP3 is significantly overexpressed in tumor breast tissues (T) compared to normal breast tissues cultured alone (N) (*p* < 0.001). in addition, a significant decrease was noticed in the expression of FOXP3 in the tumor tissues cultured in the presence of IL2 (T + IL2) compared to tumor tissues cultured alone (T) (*p* < 0.0001). On the other hand, no significant difference was noticed between FOXP3 expression in normal tissues with or without IL2 (N & N+ IL2) *p* = 0.9997 and between tumor & normal breast tissues cultured in the presence of IL2 (N+IL2 & T+IL2), *p* = 0.8433.

Regarding CTLA-4 expression, a significant difference was observed between; breast control tumor and normal tissues (T&N) *p* < 0.0001, breast normal tissues cultured without and with recombinant IL2 (N & N+IL2) *p* = 0.0069, and breast tumor tissues cultured without and with recombinant IL2 (T & T+IL2) *p* < 0.0001.

The Blimp-1 expression analysis revealed no significant difference in its expression between breast control tumor and normal tissues (T&N) *p* = 0.9770 and between breast normal tissues cultured without and with recombinant IL2 (N & N+IL2) *p* = 0.9879. Significant increase in Blimp-1 expression was noticed in breast tumor tissues cultured with recombinant IL2 as compared to the control tumor breast tissues (T+IL2 & T) *p* < 0.0001 and between tumor & normal breast tissues cultured in the presence of IL2 (N+IL2 & T+IL2) *p* < 0.0001.

#### Immunohistochemical detection of Caspase-3

**Fig. 5:**
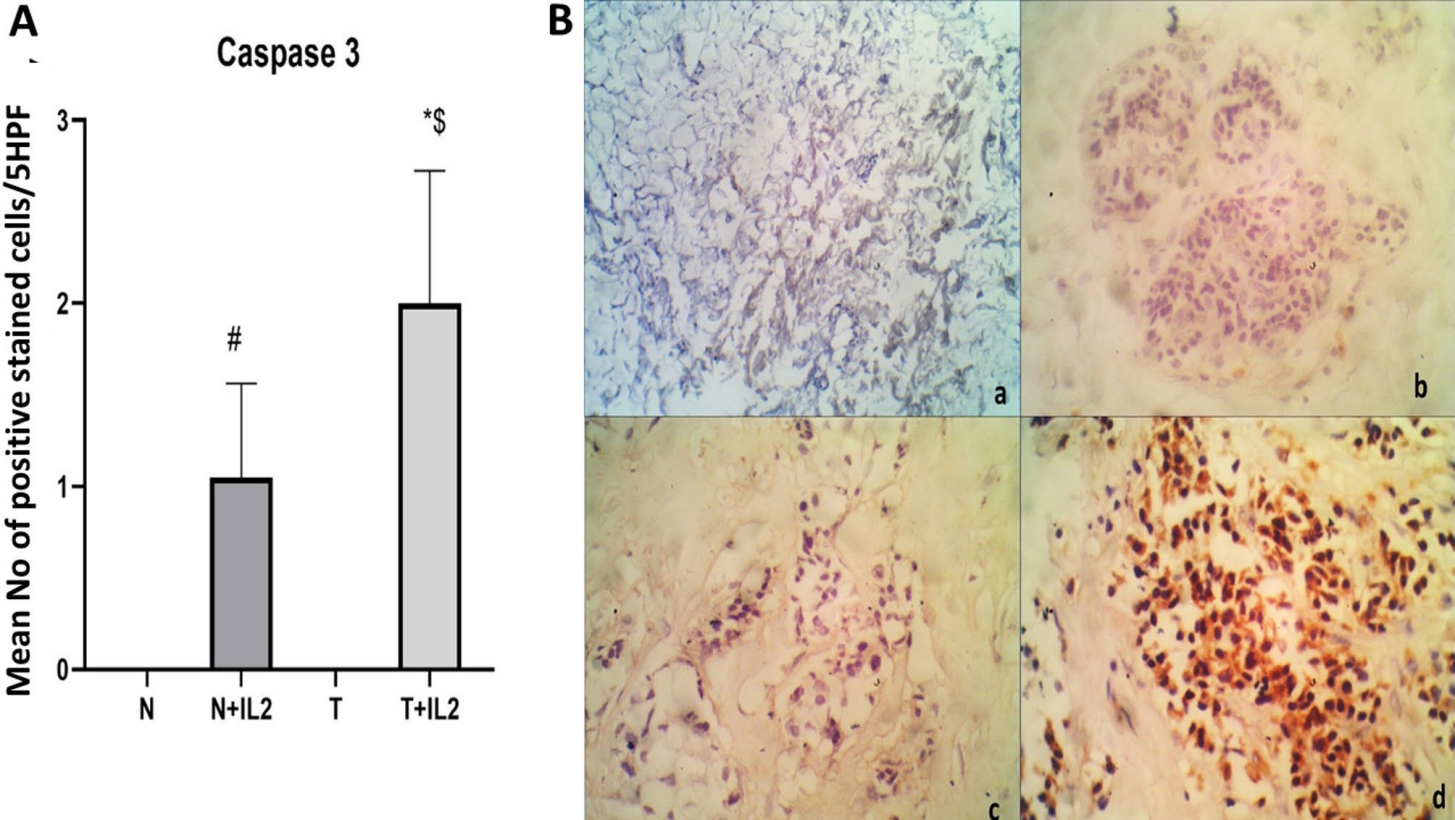
Immunohistochemical expression of caspase-3. (**A**): bar chart represents mean number of positive stained cells ± SD. # significant from N, * Significant from T, $ significant from N+IL2. (**B**): Immunohistochemical stain for caspase 3 in different groups a: Untreated normal/benign breast ducts showing negative staining of caspase- 3, (IHCX100). b: IL-2 treated normal/benign breast tissue showing negative caspase-3 staining, (IHC X400). c: IL-2 treated tumor breast tissue showing negative staining of caspase-3, (IHC X400). d: IL-2 treated tumor breast tissue showing strong positivity staining (+++) of caspase-3, (IHC X400).

**Fig. 6:**
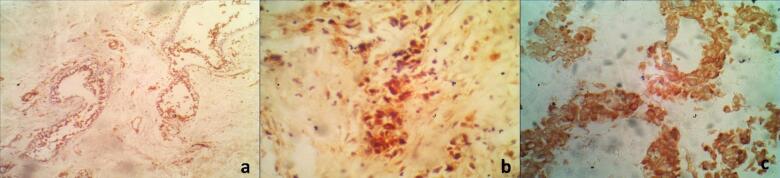
Immunohistochemical staining of caspase3 in different types of breast tumor. (**A**): IL-2 treated normal/benign breast tissues. A case of fibrocystic dilated space lined by apocrine metaplastic cells showing moderate caspase-3 staining. (IHC X100). (**B**): Il-2 treated tumor tissue. A case of invasive ductal carcinoma grade II showing strong (+++) caspase-3 staining (IHC X400). (**C**): Il-2 treated tumor tissue in a case of mucinous carcinoma. The malignant cells are surrounded by abundant extracellular mucin. IHC caspase-3 staining showing strong positivity (IHC X400).

Figures (5&6) illustrates the immunohistochemical analysis for apoptosis marker caspase-3 in the cultured breast normal and tumor tissues. There was a significant difference in the level of apoptosis between tumor tissues cultured alone and tumor tissues cultured in the presence of recombinant IL-2 (T & T+IL2), *p* < 0.0001. In addition, a significant difference was also observed between the cultured normal tissues (N& N+IL2), *p* < 0.0001 and between tumor and normal breast tissues cultured in the presence of IL-2 (N+IL2 & T+IL2), *p* < 0.0001.

#### Histopathological assessments

**Fig. 7:**
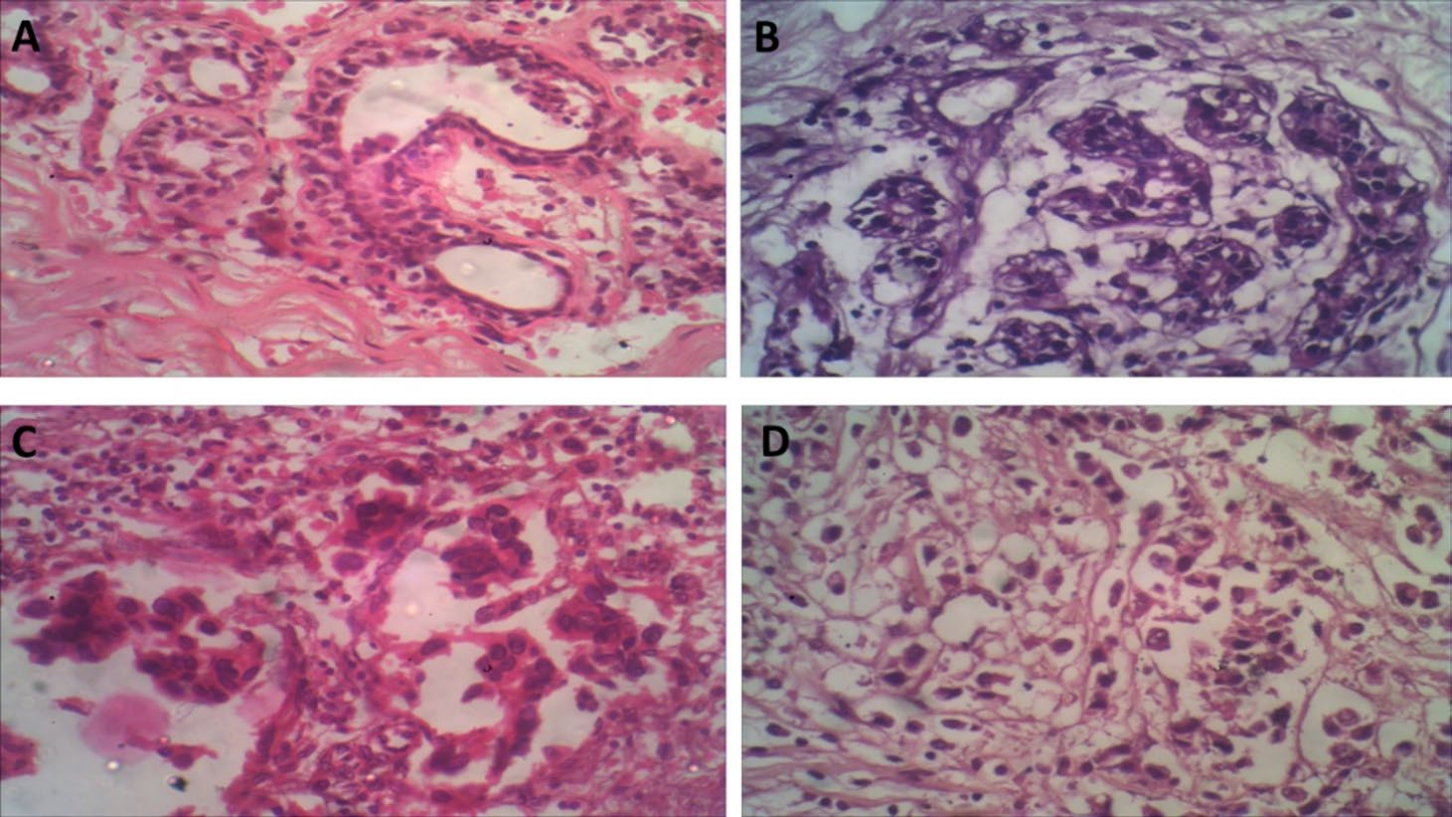
Histopathological assessment of H&E-stained sections of different groups. (**A**): Section of Untreated cultured normal/benign breast tissue showing acini lined by double layers. The inner layer is formed of cuboidal cells while the outer layer is formed of myoepithelial cells (H&E X400). (**B**): Section of IL-2 treated cultured normal/benign breast tissue showing breast lobules formed of acini lined by two layers of epithelial cells with fibromyxomatous stroma fibrous (H&E X400). (**C**): Untreated tumor tissue. A case of breast ductal carcinoma showing sheets and nests of malignant epithelial cells invading desmoplastic stroma (H&EX400). (**D**): IL-2 treated tumor tissue. A case of invasive ductal carcinoma showing cellular neoplastic growth made up of masses, dis-cohesive nests of malignant cells with abundant *eosinophilic* cytoplasm. (H&E X400).

Microscopic examination of H&E-stained slides prepared from cultured normal/benign breast tissue, showed normal breast architecture in parts of the tissue showing acini lined by double layers. The inner layer is formed of cuboidal cells while the outer layer is formed of myoepithelial cells. Other parts of tissue showed elements of fibro-cystic disease of the breast and apocrine metaplasia embedded in proliferating fibrous stroma, Figure 7 A. While, microscopic examination of H&E-stained slides prepared from IL-2 cultured treated cultured normal/benign breast tissue showed parts of normal tissue architecture along with parts showing growths made up of fibrocystic disease dilated cysts and marked adenosis, Figures 7 B.

Cultured tumor breast tissue without IL-2 treatment showed neoplastic growths made up of sheets, nests and ducts of malignant cells invading a desmoplastic stroma, markedly infiltrated by lymphocytes, Figure 7 C. IL-2 treated tumor tissue showed nests and groups of malignant ductal cells, invading desmoplastic stroma showing peri-ductal, peri-vascular and stromal moderate lymphocytic infiltration, Figure 7 D.

**Fig. 8:**
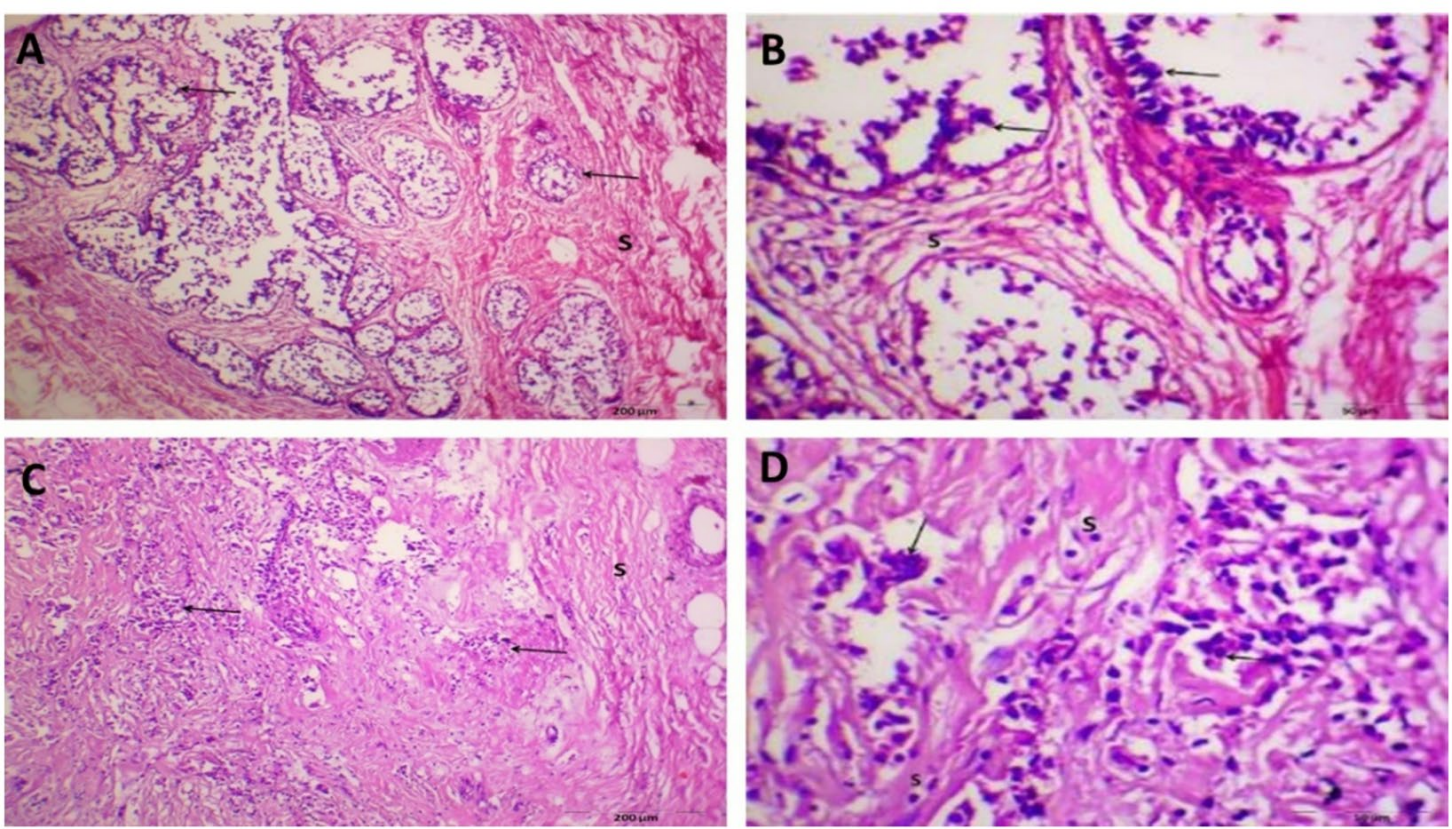
Histopathological assessment of H&E-stained sections of different groups. (**A**) A photomicrograph of section from IL-2 treated cultured breast tumor tissue in a case of invasive ductal carcinoma (Grade II) showing sheets and nests of malignant epithelial cells (↑) invading desmoplastic stroma (S). (H&E, Bar 200 μm). (**B**) High magnification of the previous figure showing cellular neoplastic growth forming nests of malignant cells (↑) with intensely chromatic nuclei and lymphocytic infiltration in the fibrous stroma (S). (H&E, Bar 50 μm) (**C**) A photomicrograph of section from IL-2 treated cultured breast tumor tissue in a case of invasive ductal carcinoma (Grade II) showing cellular neoplastic growth made up of masses, dis-cohesive nests of malignant cells in desmoplastic stroma. (H&E, Bar 200 μm). (**D**) High magnification of the previous figure showing malignant ductal cells (↑) and lymphocytic infiltration in the fibrous stroma. (H&E, Bar 50 μm).

Examined sections from IL-2 treated cultured tumor breast tissue revealed sheets and nests of malignant epithelial cells with intensely chromatic nuclei and lymphocytic infiltration in the fibrous stroma as seen in grade II of invasive ductal carcinoma, Fig. (8 A&B). Cellular neoplastic growth made up of masses and dis-cohesive nests of malignant cells as well as lymphocytic infiltration were noticed in another case of invasive ductal carcinoma with the same grade, Fig. (8C&D).

## Discussion

Breast cancer is the most commonly diagnosed malignancy worldwide with increasing rate of mortality. Breast cancer is heterogeneous due to variances in molecular subtypes, genetic alterations, hormone receptor status, and TME, all of which influence disease development, therapy response, and patient prognosis^19^. There are many challenges with the conventional treatment of breast cancer such as various adverse effects or resistance to the treatment^20^.

The **TME** of breast cancer is a highly dynamic and complex ecosystem composed of cancer cells, immune cells, stromal cells, endothelial cells, extracellular matrix components, and a variety of signaling molecules^21^. Furthermore, the **TME** of breast cancer is a critical contributing factor for tumor progression, immune evasion and immunotherapeutic resistance^1^. The anti-tumor immune response in breast cancer is mediated mainly via T cells, in the **TME**(Loi S et al.^22^,). These immune cells play a central role in recognizing and eliminating cancer cells through antigen-specific mechanisms. Among T cells, cytotoxic **CD8**^**+**^ T cells are the primary effectors of anti-tumor immunity, targeting and inducing apoptosis in tumor cells via the activation of several apoptotic pathways such as the **FAS/FASL** pathway leading to the activation of the caspase cascade and

 tumor cell apoptosis^23^.

On the other hand, tumor cells adopt several immune evasion mechanisms in order to escape the **CD8**^**+**^ T cells’ immune response via upregulation of immunosuppressive cells such as Treg cells and myeloid-derived suppressor cells (MDSCs), adipose-derived stem cells (ASCs), carcinoma-associated fibroblasts (CAFs). This finally leads to upregulation of inhibitory molecules including **CTLA-4** and **PD-1 L** which can ultimately lead to T cells apoptosis^24^. Moreover, several studies reported that **CD8**^**+**^ T cells gradually interchange toward T-cell exhaustion upon infiltration into the **TME**. T cell exhaustion is a dysfunctional state of the T lymphocytes, characterized by the downregulation of the expression of IL-2, tumor necrosis factor α (**TNF-α**), and interferon-γ (**IFN-γ**). This is accompanied by decreased proliferation, viability, upregulation of inhibitory receptor expression, and reduced effector cytolytic function (He QF et al.^25^,).

Our study aimed to assess the ex-vivo anti-breast tumor effect of IL-2 on breast cancer cells isolated from Egyptian patients after mastectomy via modulating the Treg/CTLA-4/Blimp-1/caspase-3 trajectory. But before starting the ex-vivo study, network pharmacology approach was used to identify common targets between IL-2 and breast cancer disease as well as the interconnectedness of these targets.

First, there were 35 common targets including CD4, CTLA-4, and caspase 3. The results of ex-vivo revealed a non-significant increase in the expression of **CD4** (*p* = 0.2439 and a significant increase of the co-expression of **CD4/CD25** (*p* < 0.0001) in the control tumor breast tissues compared to the normal ones was owed to the altered architecture of the TME compared to the normal breast tissues. Different studies reported that **CD4**^+^T helper cells not only increase in number within the tumor tissues but also their polarization is altered within the TME, where they changed their main subsets from Th1 in the early stages of cancer to the immune suppressive and tumor-supportive forms such as Treg and Th17 cells in the late cancer stages (Shiao SL et al.^26^,).

The Treg cells are defined as **CD4**^**+**^ T cells with a high expression of CD25 (IL-2 receptor α‐chain). Human Treg cells have been classified by Ohue and Nishikawa^27^according to expression levels of FOXP3 (and/or CD25) and a naive marker CD45RA into three fractions;1. Naive/resting Treg cells defined by FoxP3lo CD45RA+ CD25lo, 2. Effector/activated (eTreg) defined by FOXP3 hi CD45RA− CD25hi, 3. Non-Treg cells, defined by FoxP3lo CD45RA− CD25lo (Ohue Y et al.^27^,,). The Treg acquires different suppressive mechanisms to abolish CD8^+^Immune response within the TME. This includes the production of suppressive cytokines such as IL-10, TGF-β and IL-35, upregulation of checkpoints signaling including PD-1/PDL1 pathway, LAG3 pathway, and ICOS/ICOS-L signaling pathway leading to the inhibition of effector cells activation and their exhaustion within the TME (Paluskievicz CM et al.^28^,,).In addition to the direct inhibition of antigen presentation cells via the activation of CTLA-4 pathway, where it was demonstrated that activated eTregs expressing CTLA-4 receptor have the ability to bind to dendritic cells thus decreasing the expression of CD80/86 by dendritic cells hindering the activation of effector T cells within the TME^29^. Furthermore, the suppressive mechanisms of Tregs involve IL-2 signaling in several ways, including capturing IL2 within the TME by their highly expressed IL-2Rs (CD25) on Tregs thus depriving effector T cells of IL-2 that is essential for their activation leading to the suppression IL-2 transcription in effector T cells. Accordingly, high concentrations of exogenous IL-2 may directly abolish the suppressive function of Tregs^30^.

Thus, in our ex-vivo study, supplementing the TME with IL-2 within the tissue culture system significantly reduced the expression of CD4^+^/CD25^+^/FOXP3^+^ Tregs, downregulated the level of CTLA-4 expression within the TME and consequently increased the activation of effector T cells leading to induction of caspase-3 cascade and apoptosis within the breast cancer tissues.

Previous in vitro studies showed that either the exogenous addition of IL-2 or the blocking IL-2 uptake by Treg cells only and not by Teff cells – was sufficient to abrogate suppression, suggesting that IL-2 is a limiting factor for Teff cell expansion in vitro. An early predictor of suppressed Teff cell expansion in these experiments was the lack of strong IL-2Rα expression on Teff cells, accompanied reciprocally by further upregulation of IL-2Rα on Treg cells. This behavior is readily explained by competitive IL-2 consumption through Treg cells, as pSTAT5 drives IL-2Rα upregulation in both cell types^31^and^32^.

On the other hand, Li et al., showed that postoperative administration of rhIL-2 amplifies the surgery-induced augmentation of both the Treg composition and FOXP3 expression in breast cancer therapy with modified radical mastectomy highlighting the controversy regarding the effect of IL2 within the TME^33^. This heterogeneity in the IL-2 response could be owed to different factors including the pharmacogenomic aspects, where a previous study reported that polymorphism in NRAS gene significantly affects the patients’ response to high-dose IL-2, and their overall survival (Joseph RW et al^34^.).

Regarding the effect of IL-2 supplementation on the activity of CD8 + cells, it was demonstrated that Blimp-1expression is significantly upregulated in tumor cells upon IL-2 supplementation. Several studies demonstrated the role of the transcription factor Blimp-1in the activation and proliferation of CD4 + and CD8 + T cells. It was elucidated that Blimp-1is essential for the activation of CD8 + T cells to effector-killing lymphocytes but not for memory CD8 + T cells^35^. Moreover, several studies examined the role of Blimp-1 in cancer development. Blimp-1 is considered a tumor suppressor gene and its mutation is associated with different types of cancer such as B and T cell lymphomas^36^. Furthermore, Śledzińska et al., demonstrated that T-reg depletion is associated with the upregulation of Blimp-1 expression and the differentiation and activation of cytotoxic CD4 + T cells that are associated with granzyme-B production and induction of apoptosis. This polarization of cytotoxic CD4 + T lymphocytes depended mainly on IL-2 signaling^37^.

Moreover, in the current study, the levels of CD4 + CD25+ T reg cells and CD4 + cells were correlated with the clinical and pathological outcomes of the patients. We have demonstrated that the level of CD4 + cells was significantly higher in breast tumor tissue cultured with IL-2 of patients with tumor sizes less than 2 cm (T1) (*p* = 0.039) than in bigger tumor sizes and in stage I breast tumors than in later stages (*p* = 0.026). In contrast, the mean percentage of CD4 + CD25+ Tregs was significantly higher in breast tumor tissue cultured with IL-2 of patients with tumor sizes more than 2 cm (*p* = 0.039) than tumor sizes less than 2 cm and higher in later stages (*p* = 0.026) than in early breast cancer stages. According to this finding, we can also recommend early-stage breast cancer patients as a targeted group of patient populations for the current proposed IL-2-mediated therapeutic strategy.

Suggesting that the clinicopathological parameters of the patients also affect the response to IL-2 therapy, where the CD4 + cell population in the TME of early-stage breast cancer patients more effectively responded to exogenous IL-2 than those in the TME of later-stage patients. In other words, CD4 + cells like many other TILs of early-stage breast cancer patients still retain their intact signal transduction activities (IL-2/IL-2R signaling pathway).

Hence, we can propose that IL-2 mediated immunotherapeutic strategy could modulate the intra-tumoral CD4^+^ CD25^+^ T reg cells and the expression of CTLA-4 inhibitory molecules within the TME, which in turn upregulate the expression of Blimp-1 and promote the production of effector CD8^+^ T cells and cytolytic CD4^+^ T cells that transform the immune suppressive TME into immune supportive one, providing new insights relevant for the development of effective cancer immunotherapeutic approaches.

## Supplementary Information

Supplementary Information 1.

Supplementary Information 2.

Supplementary Information 3.

Supplementary Information 4.

Supplementary Information 5.

Supplementary Information 6.

## Data Availability

Data is provided within the manuscript or supplementary information files.
