# Supplementary figures and images for: Integrating network pharmacology with ex-vivo analysis to assess the effect of IL-2 in halting breast cancer: involvement of Treg/CTLA-4/Blimp-1/caspase-3
Source: Sci Rep. 2026 May 26;16:16296. doi: 10.1038/s41598-026-52551-2 (PMC13212898; doi:10.1038/s41598-026-52551-2)

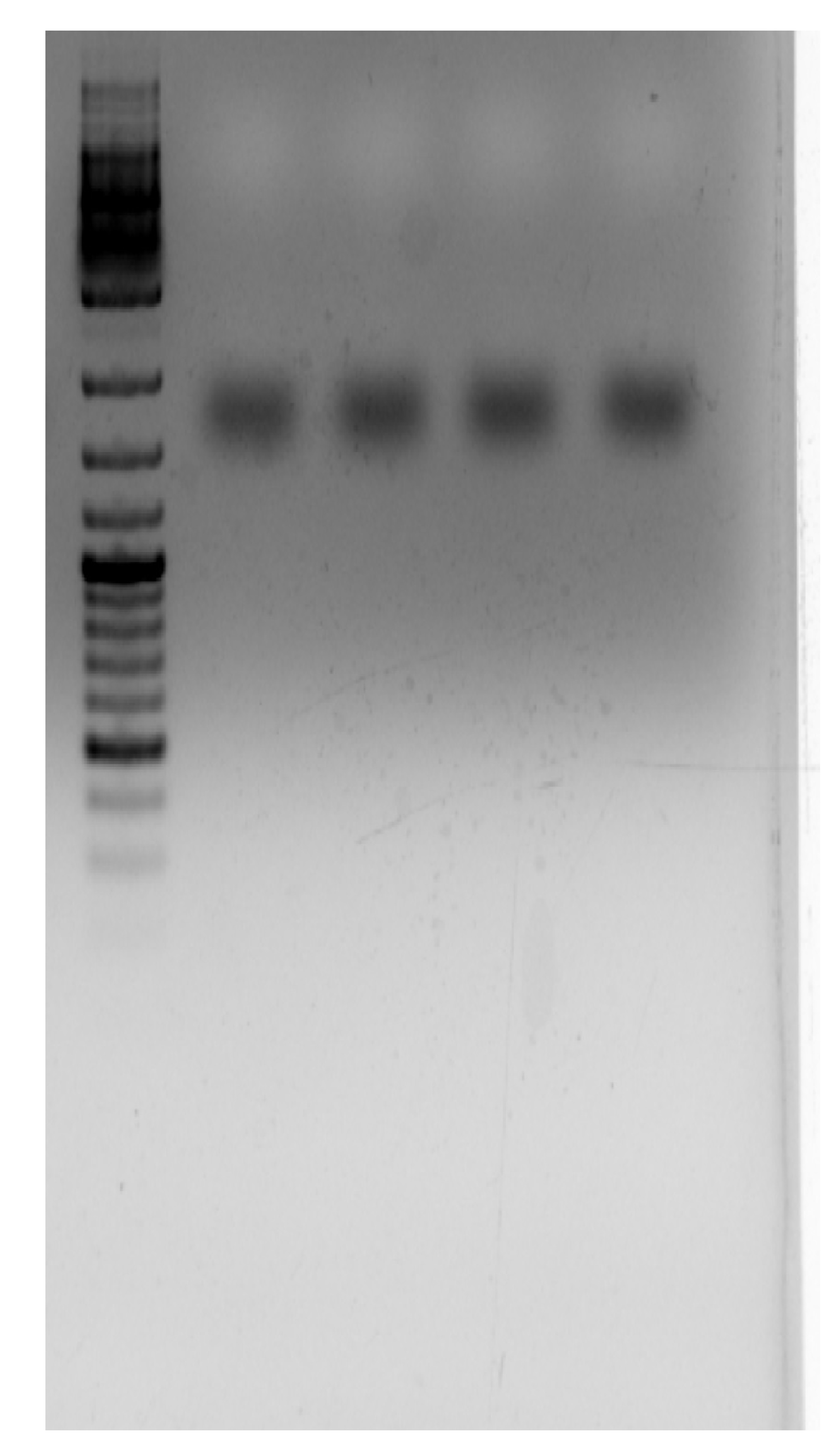

Supplement: Supplementary file 1 — Supplementary Information 1. [file 41598_2026_52551_MOESM1_ESM.jpg]

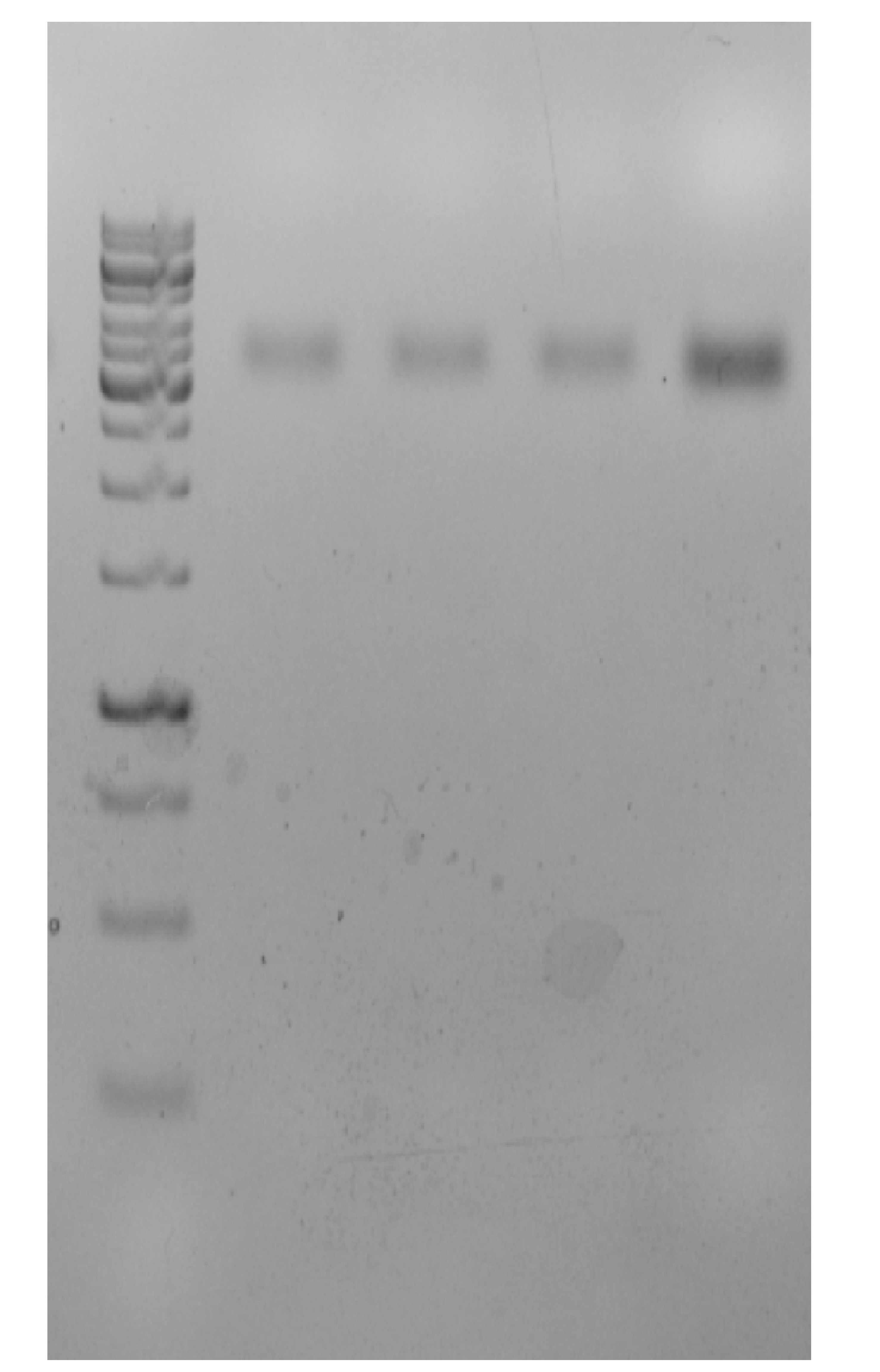

Supplement: Supplementary file 2 — Supplementary Information 2. [file 41598_2026_52551_MOESM2_ESM.jpg]

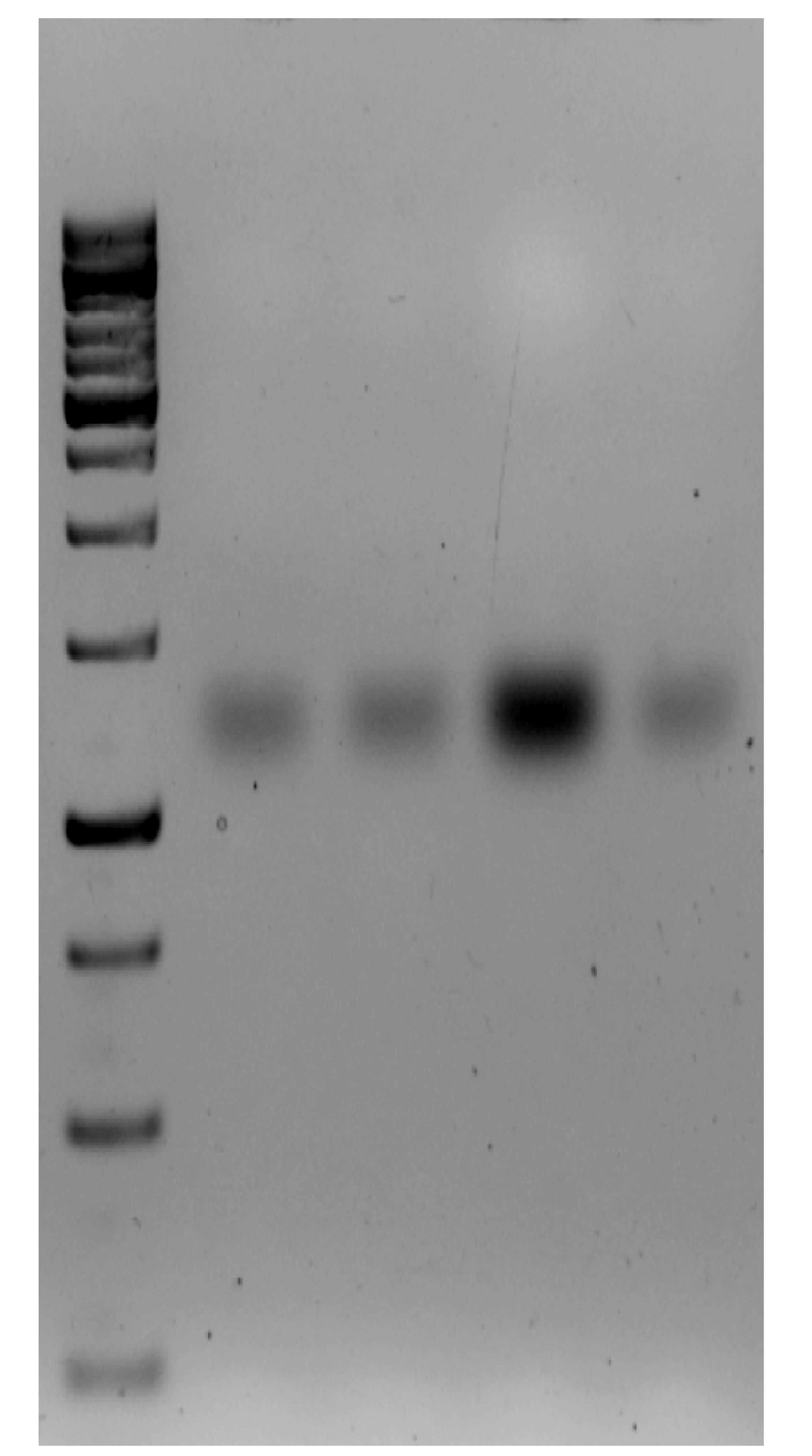

Supplement: Supplementary file 3 — Supplementary Information 3. [file 41598_2026_52551_MOESM3_ESM.jpg]

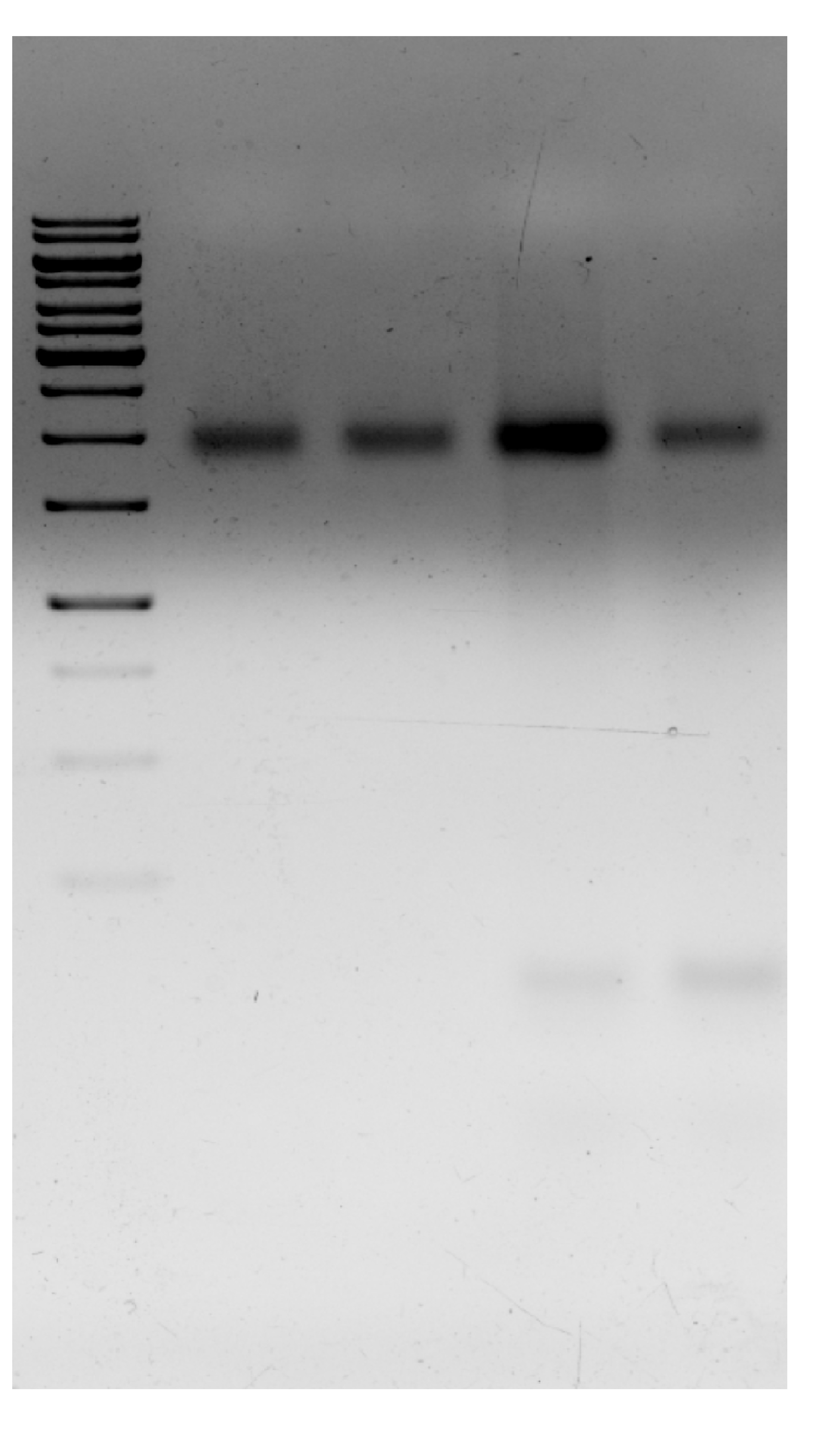

Supplement: Supplementary file 4 — Supplementary Information 4. [file 41598_2026_52551_MOESM4_ESM.jpg]
