# Supplementary material for: Integrating network pharmacology with ex-vivo analysis to assess the effect of IL-2 in halting breast cancer: involvement of Treg/CTLA-4/Blimp-1/caspase-3
Source: Sci Rep. 2026 May 26;16:16296. doi: 10.1038/s41598-026-52551-2 (PMC13212898; doi:10.1038/s41598-026-52551-2)
Supplement: Supplementary file 5 — Supplementary Information 5. [file 41598_2026_52551_MOESM5_ESM.pdf]

Alexandria University
Medical research Institute
Ethics Committee
IORG# : IORG0008812

Principle Investigator: Yasmin Mohamed Yousef Mohamed ElKabani

Protocol Title: The Effect of Interleukin-2 on Breast Cancer Tumor Microenvironment Infiltrating Regulatory T Lymphocytes.

Approval serial number: E/C. S/N. T 81/2017

The Medical Research Institute – Alexandria University Ethics Committee has reviewed the above-mentioned protocol 10/2017. Since the study guarantees the privacy of any personal information regarding the patients' data. The Alexandria University Ethics Committee granted approval for the above-mentioned study to be conducted at the Medical Research Institute – Alexandria University.

The ethics committee of the Medical Research Institute – Alexandria University is constituted and operating according to ICH GCP guidelines and applicable local and institutional regulations and guidelines which govern IRB operation.

The REC requires the submission of Safety Letters/SUSARs and Progress Reports every 6 months.

Adel

Prof. Dr. Adel Zaki

CHIEF OF ETHICAL COMMITTEE
MEDICAL RESEARCH INSTITUTE,
ALEXANDRIA UNIVERSITY

REC-MRI

165, Horreya Avenue, Hadara, Alexandria, Egypt
Tel: +203 4282331, +203 4282373 Fax: +203 4283719

Scanned with CamScanner
