# Supplementary material for: Integrating network pharmacology with ex-vivo analysis to assess the effect of IL-2 in halting breast cancer: involvement of Treg/CTLA-4/Blimp-1/caspase-3
Source: Sci Rep. 2026 May 26;16:16296. doi: 10.1038/s41598-026-52551-2 (PMC13212898; doi:10.1038/s41598-026-52551-2)
Supplement: Supplementary file 6 — Supplementary Information 6. [file 41598_2026_52551_MOESM6_ESM.pdf]

Clinico-pathological DATA

No	Age	Pathological Type	Grade	Vas. inv.	L.N inv.	Total L.N	Tumor Size	E.R	P.R	T	N	M	Stage
1	62	Invasive Ductal Carcinoma	I	-	0	9	3	+++	+++	3	0	Mx	II
2	35	Invasive Ductal Carcinoma	II	+	2	6	10	+++	++	3	1	Mx	III
3	62	Invasive Ductal Carcinoma	II	+	3	11	3	++	+++	2	1	Mx	II
4	43	Invasive Ductal Carcinoma	II	-	14	21	2	++	++	1	3	Mx	III
5	60	Invasive Ductal Carcinoma	II	+	5	7	4	+++	+++	3	2	Mx	III
6	54	Invasive Ductal Carcinoma	II	-	0	15	2،5	++	++	2	0	Mx	II
7	51	Invasive Ductal Carcinoma	III	+	0	13	3	+	+	2	1	Mx	II
8	71	Invasive Ductal Carcinoma	II	+	2	16	4	+++	++	3	1	Mx	III
9	34	Invasive Ductal Carcinoma	II	+	7	11	2،5	+++	++	2	2	Mx	III
10	64	Invasive Ductal Carcinoma	II	+	4	21	3	+++	+++	2	2	Mx	III
11	55	Invasive Ductal Carcinoma	II	+	0	12	3	+++	+++	2	0	Mx	II
12	70	Invasive Ductal Carcinoma	II	-	0	11	4	+	+	2	0	Mx	II
13	60	Mucinous carcinoma	II	+	0	9	2،5	+++	++	2	0	Mx	II
14	60	Invasive Ductal Carcinoma	II	-	1	10	3	++	++	2	1	Mx	II
15	55	Invasive Ductal Carcinoma	II	+	9	9	2	-	+	1	2	Mx	III
16	47	Invasive Ductal Carcinoma	II	+	2	11	2	+++	++	1	1	Mx	II
17	56	Invasive Ductal Carcinoma	II	+	1	10	1،5	+++	++	1	1	Mx	II

18	54	Invasive Ductal Carcinoma	II	+	0	18	2	++	++	1	0	Mx	I
19	52	Invasive Ductal Carcinoma	II	+	2	20	2	+	+	1	1	Mx	II
20	58	Invasive Ductal Carcinoma	II	+	0	12	3	++	+	2	0	Mx	II
